# Bacterial cellulose synthesis mechanism of facultative anaerobe *Enterobacter* sp. FY-07

**DOI:** 10.1038/srep21863

**Published:** 2016-02-25

**Authors:** Kaihua Ji, Wei Wang, Bing Zeng, Sibin Chen, Qianqian Zhao, Yueqing Chen, Guoqiang Li, Ting Ma

**Affiliations:** 1 Key Laboratory of Molecular Microbiology and Technology, Ministry of Education, College of Life Sciences, Nankai University, Tianjin, 300071, PR China; 2 Key Laboratory of Molecular Microbiology and Technology, Ministry of Education, TEDA Institute of Biology Sciences and Biotechnology, Nankai University, 23 Hongda Street, TEDA, Tianjin 300457, PR China; 3 Tianjin Key Laboratory of Microbial Functional Genomics, TEDA, Tianjin 300457, PR China; 4 Quality Control Department, Tsingtao Brewery Second Factory, Tsingtao Brewery CO., LTD, Qingdao 266000, PR China

## Abstract

*Enterobacter* sp. FY-07 can produce bacterial cellulose (BC) under aerobic and anaerobic conditions. Three potential BC synthesis gene clusters (*bcs*I, *bcs*II and *bcs*III) of *Enterobacter* sp. FY-07 have been predicted using genome sequencing and comparative genome analysis, in which *bcs*III was confirmed as the main contributor to BC synthesis by gene knockout and functional reconstitution methods. Protein homology, gene arrangement and gene constitution analysis indicated that *bcs*III had high identity to the *bcsI* operon of *Enterobacter* sp. 638; however, its arrangement and composition were same as those of BC synthesizing operon of *G. xylinum* ATCC53582 except for the flanking sequences. According to the BC biosynthesizing process, oxygen is not directly involved in the reactions of BC synthesis, however, energy is required to activate intermediate metabolites and synthesize the activator, c-di-GMP. Comparative transcriptome and metabolite quantitative analysis demonstrated that under anaerobic conditions genes involved in the TCA cycle were downregulated, however, genes in the nitrate reduction and gluconeogenesis pathways were upregulated, especially, genes in three pyruvate metabolism pathways. These results suggested that *Enterobacter* sp. FY-07 could produce energy efficiently under anaerobic conditions to meet the requirement of BC biosynthesis.

Bacterial cellulose (BC) is a water-insoluble extracellular polysaccharide with a simple structure. It comprises only one type of sugar (glucose), which are linked together through β-1, 4 linkages linearly. Bunches of β-1, 4 glucan chains are assembled into microfibrils in the same direction, and then crystallised into cellulose fibers^1^. BC has many excellent physicochemical properties compared with plant cellulose, such as high purity, ultrafine reticulated structure, high crystallinity, high mechanical strength, high hydrophilicity and good biocompatibility and biodegradability^2^,^3^,^4^. Consequently, BC has been widely used in the fields of paper, food, medicine, acoustic membranes, biomedical engineering and oil exploration^3^,^4^,^5^,^6^,^7^.

To date, many BC producing bacteria have been identified, including gram-negative species such as, *Gluconacetobacter xylinus*, *Achromobacter* sp., *Aerobacter* sp., *Agrobacterium* sp., *Alcaligenes* sp., *Enterobacter* sp., *Pseumdomonas* sp., *Rhizobium* sp., *Salmonella* sp. and *Sarcina* sp.; and gram-positive species, such as *Gluconoacetobacter hansenii*^8^,^9^,^10^,^11^,^12^,^13^,^14^,^15^. Among them, *G. xylinus* has been investigated extensively for its higher BC productivity^16^. The BC biosynthesis mechanisms of *G. xylinus* have been determined at the biochemical and genetic levels^17^,^18^,^19^,^20^. In *G. xylinus*, BC biosynthesis which needs precursor UDP-glucose (UDPG) and activator cyclic diguanylic acid (c-di-GMP) is accomplished by a cellulose synthase complex containing BcsA, BcsB, BcsC and BcsD subunits, which are encoded by genes within a single operon^21^,^22^,^23^,^24^. BcsA and BcsB form the minimum complex required for cellulose synthesis^23^,^25^. BcsA containing eight transmembrane helices, is the catalytic subunit of cellulose synthase complex, and belongs to the GT-2 glycosyltransferase family^25^,^26^. BcsB is an auxiliary subunit that interacts with BcsA via its C-terminal transmembrane helix and regulates cellulose synthesis by interacting with c-di-GMP. Wong *et al.* proved that BcsC is essential for cellulose synthesis *in vivo*, but not required for β-1, 4 glucan synthesis *in vitro*^27^. Saxena *et al.*^28^ suggested that the BcsC subunit might form a pore in the cell membrane for cellulose secretion using bioinformatic analysis. Mutants with an interrupted *bcs*D gene showed 40% decreased cellulose production, but produced both cellulose I and II, suggesting that BcsD controls the crystallisation of cellulose into nanofibrils^27^,^28^. The structure of BcsD showed an exquisite cylinder shape with a right-hand twisted dimer on the cylinder wall and formed a functional octamer unit suggesting that BcsD could provide a passageway for extruding the glucan chains^29^. UDP-glucose pyrophosphorylase (UGPase) which is encoded by *galU*^30^,^31^ converts glucose-1-phosphate (Glc-1-p) to UDPG. Phosphoglucomutase (PGM) which is encoded by *pgm* gene converts glucose-6-phophate to Glc-1-p^32^,^33^. PGM bridges the BC polymerisation steps with the common cellular metabolite Glc-6-P^34^. As an allosteric activator of cellulose synthase, c-di-GMP is essential to activate cellulose synthase^25^,^35^. The c-di-GMP activator is synthesised from GTP by diguanylate cyclase, and degraded by two phosphodiesterases (PDE-A and PDE-B) to regenerate 5′GMP, which may be used for GTP synthesis and completes the biosynthetic and degradative pathway cycle of c-di-GMP^36^,^37^. In addition to the above genes, cellulose synthesis-related genes are located both upstream and downstream. In the most well studied strain *G. xylinus* ATCC 53582, the upstream region contains the *cmcax* and *ccpax* genes^38^,^39^. Endo-β-1, 4-glucanase encoded by gene *cmcax* has cellulose hydrolysing activity and can enhance cellulose synthesis^40^. The CcpAx protein is essential for cellulose production and plays a critical role in locating the cellulose synthesising complex on the cell membrane^38^. Sunagawa *et al.* suggested that the CcpAx protein functions as a mediator of protein-protein interactions^41^. The *bglxa* gene is located downstream of the BC synthesis operon and encodes a β-glucosidase; disruption of *bglxa* causes a decrease in cellulose production^21^.

Despite being a well-studied strain for BC biosynthesis research, *G. xylinus* is a strict aerobe that can only produce BC under aerobic conditions^42^. BC production of *G. xylinus* can only be accomplished through tray fermentation and is costly. Recently, our lab reported an *Enterobacter* sp. FY-07 that can produce BC under aerobic and anoxic conditions^12^. *Enterobacter* sp. FY-07 has very closely evolutionary relationship with *Enterobacter* sp. 638. Although possess two BC synthesis operon, *Enterobacter* sp. 638 only produce a little amount of BC as a component of biofilm^43^. To identify the BC synthesis gene cluster of *Enterobacter* sp. FY-07, comparative genomic analysis, gene knockout and functional reconstitution methods were performed. To investigate whether the different oxygen demands lead to the different BC synthesis mechanisms between *G. xylinus* ATCC 53582 and *Enterobacter* sp. FY-07 or why *Enterobacter* sp. FY-07 has the different BC production ability with *Enterobacter* sp. 638, we compared the characteristics of the BC synthesis gene cluster among *Enterobacter* sp. FY-07, *G. xylinus* ATCC 53582 and *Enterobacter* sp. 638. To clarify the differences in BC biosynthesis of *Enterobacter* sp. FY-07 under aerobic and anaerobic conditions, comparative transcriptome analysis and metabolite quantitative experiments were performed.

## Results

### Genome sequencing of *Enterobacter* sp. FY-07

**Figure 1:**
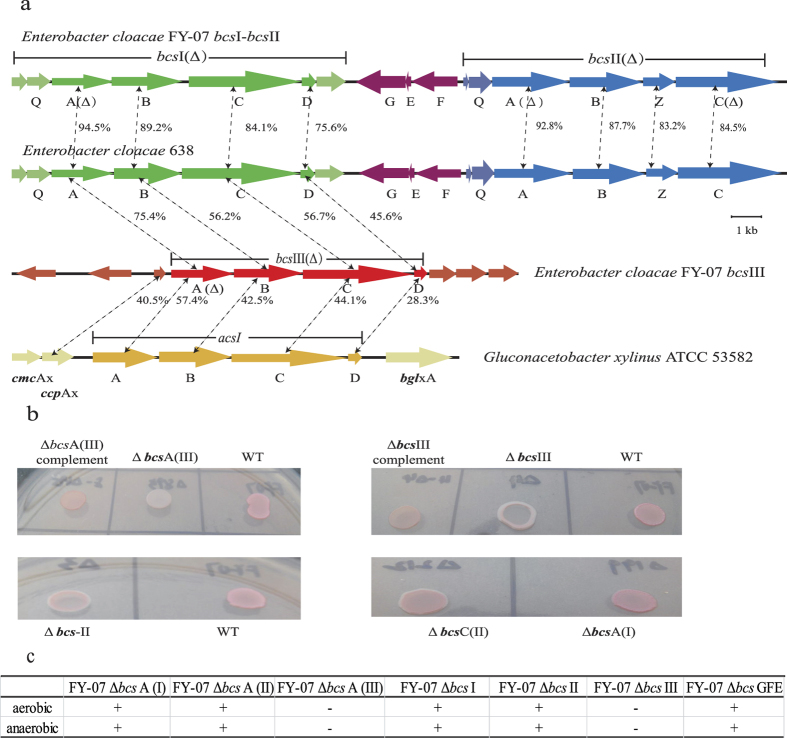
Cellulose synthesis-related gene clusters from *Enterobacter* sp. FY-07. (**a**) Structure of cellulose synthesis-related gene clusters from *Enterobacter* sp. FY-07, *Enterobacter* sp. 638 and *G. xylinum* ATCC 53582; (**b**) Effects of gene-knockout on the cellulose production of *Enterobacter* sp. FY-07. (**c**) The bacterial cellulose (BC) production ability of *Enterobacter* sp. FY-07 gene knockout mutants under aerobic and anaerobic conditions.

Although a few BC-producing *Enterobacter* sp. have been reported^11^,^12^,^16^, there has been no report of their gene information and cellulose synthetic mechanism. The genome of *Enterobacter* sp. FY-07 is the first sequenced genome of an *Enterobacter* sp. strain which is characterized as highly effective BC producer. The genome of *Enterobacter* sp. FY-07 consists of one 5.12 Mbp chromosome (Genbank access number: CP012487) and three plasmids, pAKI40A (CP012488), pAKI40B (CP012489) and pAKI40C (CP012490) of 2324 bp, 3776 bp and 13955 bp, respectively. The GC content of the chromosome is 53.62%, whereas the three plasmids have GC contents of 51.81%, 44.65% and 56.47%. Overall 5019 “locus_tags” were identified in the chromosomal sequence, among which 4916 are protein-encoding genes, 82 are tRNA-encoding genes, and 21(organized in 7 operons) are rRNA-encoding genes. From practical point of view, BC production is the most important feature of the *Enterobacter* sp. FY-07. Three bacterial cellulose synthase operons (*bcs*) designated as *bcs*I, *bcs*II and *bcs*III were identified in the genome using comparative genome analysis (Fig. 1a). All three are structurally complete. Operon *bcs*I comprises seven genes encoding: hypothetical protein (AKI40_0196); YhjQ (AKI40_0197); four bacterial cellulose synthase subunits: BcsA (AKI40_0198), BcsB (AKI40_0199), BcsC (AKI40_0200) and BcsD (AKI40_0201); and cellulase (AKI40_0202). Operon *bcs*II comprises six genes: *yhj*R (AKI40_0206), *yhj*Q (AKI40_0207), *bcs*A (AKI40_0208), *bcs*B (AKI40_0209), *bcs*Z (AKI40_0210) and *bcs*C (AKI40_0211). Operon *bcs*III only comprises four cellulose synthase subunits encoding genes: *bcs*A (AKI40_0894), *bcs*B (AKI40_0893), *bcs*C (AKI40_0892) and *bcs*D (AKI40_0891). In addition, there is an operon with three genes in the opposite orientation between operon *bcs*I and operon *bcs*II. These three genes are *bcs*G (AKI40_0203), *bcs*F (AKI_0204) and *bcs*E (AKI_0205), which are considered related to bacterial cellulose synthesis.

### Identification of operons and genes responsible for BC production in *Enterobacter* sp. FY-07

Gene knockout, gene complementation and functional reconstitution methods were used to determine which operon is essential for BC synthesis and whether this operon plays the same role in BC synthesis in *Enterobacter* sp. FY-07 under aerobic and anaerobic conditions. Seven gene knockout mutants were constructed and designated as FY-07 Δ*bcs*I; FY-07 Δ*bcs*II; FY-07 Δ*bcs*III; FY-07 Δ*bcs*GFE; FY-07 Δ*bcs*A (I); FY-07 Δ*bcs*A (II) and FY-07 Δ*bcs*A (III). The locations of the gene knockouts are shown in Fig. 1a. Only the *bcs*III-knockout mutants and the *bcs*A (III)-knockout mutants produced no cellulose under aerobic and anaerobic conditions (Fig. 1b,c). When a plasmid harboring the native *bcs*A or *bcs*III was introduced into the corresponding mutant, the complemented strains recovered their BC production ability (Fig. 1b). The function of *bcs*III operon was also proven using functional reconstitution experiment. The bcsIII operon was amplified and inserted between the *EcoR*I and *Hind*III sites of the pBAD30, yielding the *bcs*III-expressing vector. The vector was transformed into *E. coli* DH5α, yielding *E. coli* DH5α (pBABIII).The functional reconstitution strain *E. coli* DH5α (pBABIII) can produce BC when induced by 0.2% L-arabinose (supplementary Fig. S1b). These results confirmed that the operon *bcs*III is essential for cellulose production of *Enterobacter* sp. FY-07. To investigate whether the different oxygen demands lead to the different BC synthesis mechanisms between *G. xylinus* ATCC 53582 and *Enterobacter* sp. FY-07 or why *Enterobacter* sp. FY-07 has the different BC production ability with *Enterobacter* sp. 638, the protein homology, arrangement and constitution of the genes involved in the cluster were compared among *Enterobacter* sp. FY-07, *Enterobacter* sp. 638 and *G. xylinum* ATCC 53582. Protein sequence alignment was performed at the EMBL-EBI website (http://www.ebi.ac.uk/Tools/psa/) (Fig. 1a). The gene constitution and arrangement of *Enterobacter* sp. FY-07 BC synthesis gene cluster *bcs*III, comprising *bcs*A, *bcs*B, *bcs*C and *bcs*D, are same as *G. xylinum* ATCC53582 but has much higher homology with *Enterobacter* sp. 638 than with *G. xylinum* ATCC53582, suggesting that the BC synthesis mechanism of *Enterobacter* sp. FY-07 is same to those of *G. xylinum* ATCC53582 and there exists limiting factor for BC production in *Enterobacter* sp. 638.

### BC biosynthesis under aerobic and anaerobic conditions

**Figure 2:**
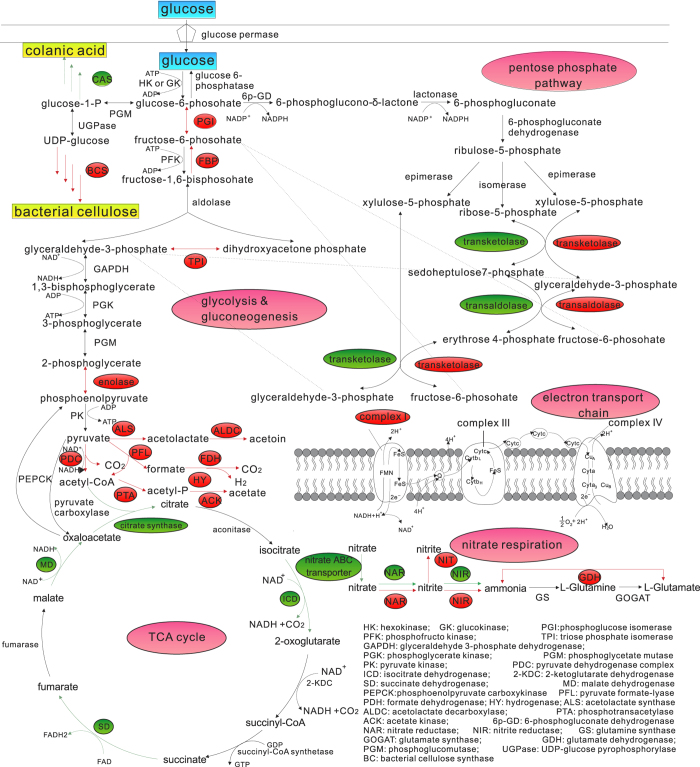
Transcription profiles of genes encoding enzymes for the glycolytic pathway, gluconeogenic pathway, pentose phosphate pathway, TCA cycle, nitrate reduction pathway, bacterial cellulose synthesis pathway and colanic acid synthesis pathway. Green indicates that the transcription of the gene encoding the enzyme related to this reaction was downregulated under anaerobic conditions. Red indicates upregulated under anaerobic conditions.

We performed comparative transcriptome analysis to investigate whether BC biosynthesis by *Enterobacter* sp. FY-07 is in the same way under aerobic and anaerobic conditions. According to the reported BC biosynthesis pathway and protein homology analysis, the BC biosynthesis pathway of *Enterobacter* sp. FY-07 includes the following four reactions^26^. Firstly, glucose and ATP are converted to glucose-6-phosphate and ADP by glucokinase (GK). Secondly, glucose-6-phosphate is catalysed by PGM to glucose-1-phosphate. Then, UTP and glucose-1-phosphate are catalysed to UDPG and ppi by UGPase. Finally, UDPG is polymerised to BC by a BC synthase complex encoded by the *bcs*III operon (Fig. 2). The polymerisation needs c-di-GMP as an activator. On the basis of protein homology analysis, GK, PGM and BC synthase complex are encoded by AKI40_1504, AKI40_2159, and AKI40_0894~AKI40_0891 respectively. Protein homology of GK and PGM is 96.6% and 97.3% to corresponding orthologs in *Enterobacter* sp. 638 (ENT638_RS15075; ENT638_RS06320). UGPase can be encoded by three gene copies: AKI40_0889, AKI40_1747, and AKI40_3354; the protein homologies of the UGPases are 68.6%, 97% and 96.1% to corresponding orthologs in *Enterobacter* sp. 638 (ENT638_RS11900, ENT638_RS13725), respectively. Comparative transcriptome results also clearly illustrated that the same BC biosynthesis pathway was employed by *Enterobacter* sp. FY-07 under aerobic and anaerobic conditions. Transcriptome sequencing results have been submitted to NCBI and the SRA number of samples under aerobic and anaerobic conditions is SRR2183388 and SRR2183389, respectively. Transcription of *Enterobacter* sp. FY-07 genes encoding GK, PGM and UGPase was essentially the same under aerobic and anaerobic conditions (supplementary Table S2). In addition, transcription of BC biosynthesis genes was analysed. The results showed that gene transcription of operon *bcs*III provided an absolute advantage under aerobic and anaerobic conditions and was consistent with the results of the gene knockout experiments (supplementary Table S2).

### Energy metabolism

Analysis of the BC biosynthesis process of *Enterobacter* sp. FY-07 suggested that there is no oxygen included in these reactions directly; however, adenosine triphosphate (ATP) is required to activate glucose to UDPG. In addition, ATP is also essential for the biosynthesis of c-di-GMP, which is the activator of BC biosynthesis. Therefore, BC biosynthesis of *Enterobacter* sp. FY-07 is not directly related to oxygen but is related to energy production under aerobic and anaerobic conditions. To investigate how *Enterobacter* sp. FY-07 ensures a sufficient ATP supply for BC biosynthesis under aerobic and anaerobic conditions and how *Enterobacter* sp. FY-07 produces a similar yield of BC under aerobic and anaerobic conditions, transcriptome sequencing was performed to analyse the energy production modes under aerobic and anaerobic conditions. Figure S2 provides a summary of the percentage of differentially expressed genes grouped by functional categories. Under anaerobic conditions, genes related to amino acid transport and metabolism (E), cell wall/membrane biogenesis (M), translation (J), energy production and conversion (C) showed higher-levels of expression as compared to under aerobic conditions. In the fermentation culture conditions, glucose catabolism is the mainly energy source of *Enterobacter* sp. FY-07 under aerobic and anaerobic conditions. The production of ATP has two patterns: substrate-level phosphate through glucose metabolism; and oxidative phosphate through NADH (sometimes FADH_2_) produced by glucose metabolism transferred to electron acceptors.

The transcription for glucose metabolism, electron transport chain and nitrogen metabolism related genes were analysed under aerobic and anaerobic conditions (Fig. 2). Five genes encoding for three glycolytic pathway key enzymes, GK (AKI40_1504), 6-phosphofructokinase (AKI40_4866; AKI40_2744) and pyruvate kinase (AKI40_2849; AKI40_3571) were not differentially expressed under anaerobic conditions (Fig. 2 and supplementary Table S2). The results indicated that the glycolytic pathway was not differentially regulated under anaerobic conditions. However, genes encoding for glucose-6-phosphate isomerase (AKI40_4682), triosephosphate isomerase (AKI40_4862) and enolase (AKI40_4050), were upregulated under anaerobic conditions. Upregulation of the three genes and the genes encoding for fructose-1, 6-bisphosphatase (AKI40_1324; AKI40_4472) suggested the activation of gluconeogenesis pathway under anaerobic conditions (supplementary Table S2). Gene (AKI40_3569) encoding for glucose-6-phosphate dehydrogenase which is the rate-limiting enzyme of the pentose phosphate pathway was not differentially expressed under anaerobic conditions. The results indicated that the PPP metabolism did not change significantly under anaerobic conditions. The differentially expression of transketolase genes (AKI40_0825, AKI40_1411) and transaldolase (AKI40_4259, AKI40_1412) genes suggested that different genes contributed to reversible conversion among hexoses, pentoses and tetroses under aerobic and anaerobic conditions. Most TCA cycle genes, such as those encoding citrate synthase (AKI40_2176), isocitrate dehydrogenase (AKI40_2659), succinate dehydrogenase (AKI40_2177, AKI40_2179, AKI40_ 2180) and malate dehydrogenase (AKI40_0551, AKI40_3010) were downregulated, suggesting the repression of TCA cycle in *Enterobacter* sp. FY-07 under anaerobic conditions(Fig. 2 and supplementary Table S2). Transcriptions of genes encoding the key enzymes isocitrate lyase (AKI40_4699) and malate synthase (AKI40_4700) of the glyoxylate pathway were expressed at very low levels under aerobic and anaerobic conditions (supplementary Table S2), suggesting that although *Enterobacter* sp. FY-07 possesses genes for this metabolism, glyoxylate metabolism is inactivated.

Compared with aerobic conditions, the glycolytic pathway was not significantly changed, but the rate of TCA cycle was reduced under anaerobic conditions. Thus, pyruvate, as the final product of the glycolytic pathway, cannot be consumed by the TCA cycle. The results, combined with transcriptome sequencing and quantitative PCR analysis, suggested that *Enterobacter* sp. FY-07 possesses three significantly upregulated pyruvate metabolic pathways under anaerobic conditions (Fig. 2 and supplementary Table S2). The first pathway is the phosphotransacetylase-acetate kinase (Pta-AckA) pathway, which consists of three enzymatic reactions. Genes of the pathway were upregulated under anaerobic conditions. In this pathway, pyruvate is oxidised by the pyruvate dehydrogenase complex (AKI40_4164~AKI40_4167) or formate-lyase (AKI40_2388), resulting in the formation of acetyl coenzyme A. The conversion of acetyl-CoA to acetic acid is catalysed by acetyltransferase (AKI40_1562) and acetate kinase (AKI40_1564), and couples with substrate-level ATP production. The second pathway is the formate-lyase pathway, with formic acid as the intermediate metabolite, H_2_, CO_2_ and NADH as the end-products. Genes of this pathway encoding formate-lyase (AKI40_2388), formate dehydrogenase (AKI40_3156~AKI40_3158, AKI40_3242, AKI40_4599) and hydrogenase enzymes (AKI40_0155, AKI40_0754~AKI40_0761, AKI40_1035) were upregulated under anaerobic conditions. The third pathway is the acetoin biosynthesis pathway. Pyruvate is catalysed by acetolactate synthase (AKI40_0023~AKI40_0024, AKI40_4203~AKI40_4204, AKI40_4818, AKI40_0779), resulting in the synthesis of acetolactate. The transformation of acetolactate to acetoin is catalysed by acetolactate decarboxylase (AKI40_0778). Transcriptions of the acetolactate synthase gene and the acetolactate decarboxylase gene were upregulated under anaerobic conditions. In addition, transcription of genes encoding alcohol dehydrogenase and lactate dehydrogenase were not regulated under anaerobic conditions. These results suggested that lactate fermentation and ethanol fermentation do not exist in *Enterobacter* sp. FY-07 under anaerobic conditions. The regulation of genes involved in the above pathways was confirmed by qRT-PCR analysis (supplementary Table S2).

**Figure 3:**
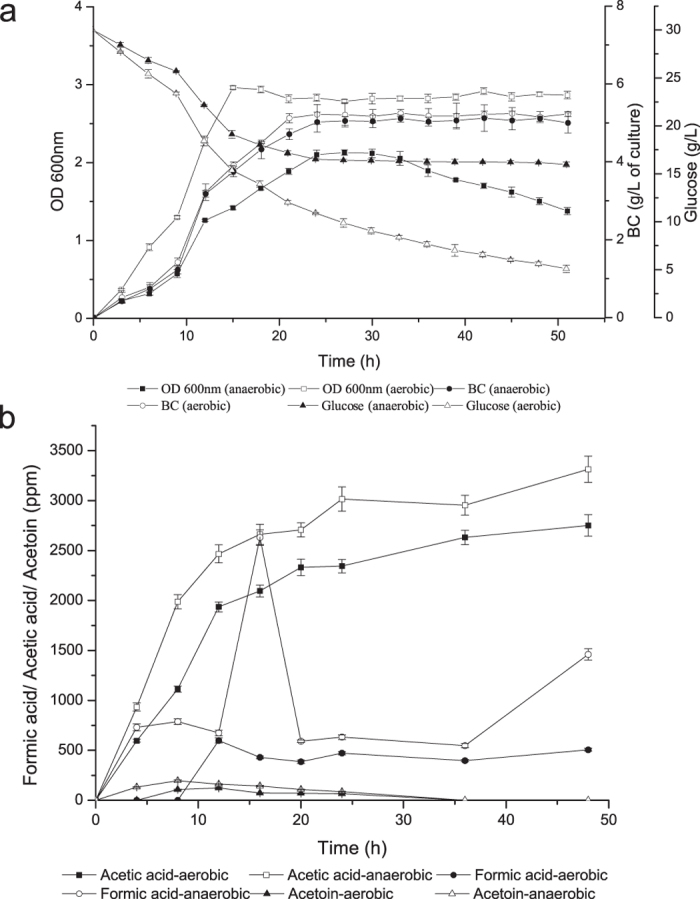
(**a**) Cell growth, BC production and glucose comsumption of *Enterobacter* sp. FY-07. (**b**) Metabolite quantitative analysis of *Enterobacter* sp. FY-07 in fermentation medium under aerobic and anaerobic conditions. Data indicate the means ± standard deviations from three independent experiments performed in triplicate.

To better understand the growth and metabolism of *Enterobacter* sp. FY-07 under aerobic and anaerobic conditions, we measured the cell growth, BC yield, and the concentrations of glucose, formic acid, acetic acid, acetoin, lactic acid and ethanol in the culture broth (Fig. 3). The BC production of *Enterobacter* sp. FY-07 is similar under aerobic and anaerobic conditions (Fig. 3a). As shown in Fig. 3b, the concentration of acetic acid and formic acid under anaerobic conditions were higher than those under aerobic conditions. In addition, a higher concentration of acetoin was detected under anaerobic conditions than aerobic conditions. The other fermentation end-products such as lactic acid and ethanol were not detectable under aerobic and anaerobic conditions. Metabolite quantitative analysis results were basically consistent with the results of transcriptome sequencing. The results suggested that glucose was metabolised by the glycolytic pathway, pyruvate metabolism and TCA cycle, with carbon dioxide (CO_2_) as the main final metabolite of *Enterobacter* sp. FY-07 under aerobic conditions. Under anaerobic conditions, glucose was incompletely metabolised by the glycolytic pathway and pyruvate metabolism to form more formic acid, acetic acid, acetoin and hydrogen than aerobic conditions as the products.

### Electron transfer

Oxygen is the electron acceptor under aerobic conditions. Electrons of NADH and FADH_2_, generated from the glycolytic pathway and TCA cycle, are transferred to oxygen through the electron transfer chain. Energy production is mainly based on oxidative phosphorylation, supplemented by substrate-level phosphorylation under aerobic conditions^44^. The glycolytic pathway was not differentially regulated, but the rate of TCA cycle was repressed under anaerobic conditions. However, only a few ATP molecules are produced by glycolytic pathway. To ensure the energy production for BC biosynthesis, the respond of *Enterobacter* sp. FY-07 to anaerobic conditions was studied. According to the transcriptome sequencing and qRT-PCR analysis, transcriptions of genes encoding alcohol dehydrogenase (AKI40_3245, AKI40_3351, and AKI40_4427) and lactate dehydrogenase (AKI40_1686) were not differentially express under anaerobic conditions, suggesting that the NADH produced by the glycolytic pathway cannot be re-oxidised by fermentation. The substance functioning as the electron acceptor under anaerobic conditions was identified using transcriptome sequencing. Transcriptions of genes in nitrogen metabolism encoding dissimilatory nitrate reductase (AKI40_3370~AKI40_3367), nitrite reductase (AKI40_0420, AKI40_0421), nitrite transporter (AKI40_0419) were upregulated under anaerobic conditions (Fig. 2 and supplementary Table S2), proving that nitrate is the electron acceptor under anaerobic conditions. Nitrate accepting the electrons transported through the electron transporter chain is reduced to nitrite. Nitrite is then reduced to ammonia. The results suggested that substrate-level phosphorylation and oxidative phosphorylation are both energy production modes under anaerobic conditions. In addition, transcriptions of genes encoding assimilative nitrate reductase (AKI40_1028, AKI40_1029) were downregulated under anaerobic conditions. These results suggested that *Enterobacter* sp. FY-07 has different pathways for nitrate utilisation under aerobic and anaerobic conditions. *Enterobacter* sp. FY-07 undergoes nitrate respiration under anaerobic conditions. Anaerobic nitrate respiration is an effective energy production mode and can supply energy for BC biosynthesis. All the results were confirmed by qRT-PCR analysis (supplementary Table S2).

**Figure 4:**
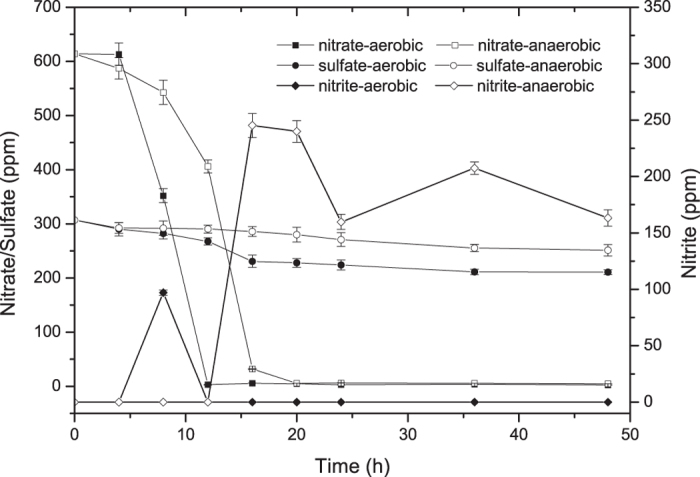
Summary of *Enterobacter* sp. FY-07 cultures investigated in this study. Nitrate, nitrite and sulfate concentrations in the culture medium are shown. Values are the means of three experiments with standard deviations.

In order to verify the results, the concentration of sulfate, nitrate and nitrite in the medium were determined under aerobic and anaerobic conditions (Fig. 4). The concentration of sulphate decreased slightly under aerobic and anaerobic conditions. However, nitrate was consumed rapidly under aerobic and anaerobic conditions, being consumed completely at 12^th^ hour and 20^th^ hour respectively. Although it was not added to the culture, a large amount of nitrite was detected in the culture medium after 16^th^ hour under anaerobic conditions. Nitrite was only detected at 8^th^ hour under aerobic conditions. These results agreed with the upregulation of transcription of gene (AKI40_0419) encoding nitrite transporter under anaerobic conditions. All these results provided direct evidence for nitrate being the electron acceptor of *Enterobacter* sp. FY-07 under anaerobic conditions.

## Discussion

In this study, the BC biosynthesis genes cluster of *Enterobacter* sp. FY-07 was identified using gene knockout, gene complementation methods and functional reconstitution experiment. *bcs*III operon is the functional BC biosynthesis gene cluster under aerobic and anaerobic conditions. Protein sequence alignment proved that genes constitution and arrangement of *bcs*III operon are the same as BC synthesis operon of *G. xylinus* ATCC 53582. The result suggested the same BC synthesis mechanism of *Enterobacter* sp. FY-07 as *G. xylinus* ATCC 53582. However, the flanking sequences of *Enterobacter* sp. FY-07 BC synthesis gene cluster are totally different from those of *G. xylinum* ATCC53582^20^ (Fig. 1a). The gene (AKI40_0895) upstream of *bcs*A (III) in *Enterobacter* sp. FY-07 encodes a hypothetical protein. When knocked out, the BC biosynthesis ablility of mutant *Enterobacter* sp. FY-07 (Δ*hyp*) decreased sharply (supplementary Fig. S1). In *G. xylinum* ATCC 53582, the gene in the location corresponding to AKI40_0895, encodes a cellulose complementing factor (CcpAx)^41^, which was essential for BC production. Although its function in BC synthesis remains unknown, the absence of the CcpAx protein causes a lack of BC production ability. Protein sequence alignment proved that there is very low similarity between the unknown function protein and CcpAx (40.5%) (Fig. 1a). The genes (AKI40_0896 and AKI40_0897) encoding hypothetical protein and diguanylate cyclase exist upstream of the AKI40_0895 gene in *Enterobacter* sp. FY-07. However, in *G. xylinum* ATCC 53582 the gene *cmcAx* encoding a cellulase, is upstream of *ccpax*^21^. Downstream of the BC synthesis genes cluster in *Enterobacter* sp. FY-07 are genes (AKI40_0890, AKI40_0889, AKI40_0888) encoding diguanylate cyclase/phosphodiesterase, UTP-glucose-1-phosphate uridylyltransferase and a minor endoglycanase Y; however, in *G. xylinum* ATCC 53582 there is gene encoding β-glucosidase^21^. Sequence comparison revealed that the genetic constitution and arrangement of operons *bcs*I and *bcs*II are the same as those in *Enterobacter* sp. 638 (Fig. 1a). However, in *Enterobacter* sp. 638 there is no gene cluster similar to *bcs*III. This is why *Enterobacter* sp. FY-07 produces large clumps of cellulose, but only a small amount of BC is produced as a component of the biofilm by *Enterobacter* sp. 638^43^. The high homology between *Enterobacter* sp. FY-07 *bcs*III cluster and *Enterobacter* sp. 638 *bcs*I cluster suggested that *Enterobacter* sp. 638 also possess the BC biosynthesis ability. The transcriptions of *bcs*I and *bcs*II were very low in *Enterobacter* sp. FY-07 (supplementary Table S2). These results suggested that some limiting factors of the transcription of *bcs*I and *bcs*II cluster exist. Regulation of *bcs*I and *bcs*II cluster needs to be further studied. In *E. coli* 1094, *bcs*Q which also exists in the *bcs*I and *bcs*II cluster, is an essential component of the cellulose synthesis apparatus that localises at the bacterial cell pole^45^. In this study, knockout of clusters *bcs*I or *bcs*II did not affect the BC biosynthesis capacity in *Enterobacter* sp. FY-07. The functions of other genes, such as *bcs*F, *bcs*E and *bcs*G, have not been identified. *Enterobacter* sp. FY-07Δ*bcs*GFE retained the ability to synthesise BC.

According to the comparative transcriptome and metabolite quantitatively analyze, most of the pyruvate which cannot enter TCA cycle was used for producing a large amount of formic acid, acetic acid and a small amount of acetoin rather than lactic acid and ethanol under anaerobic conditions.The formation of acetic acid was coupled with ATP biosynthesis. Under anaerobic conditions, the concentration of formic acid was increased first and then reduced in the culturing process. This may be due to that formic acid is the intermediate metabolite and can be further utilized in the culturing process^46^. The further metabolism of formic acid is catalysed by formate dehydrogenase and couples with the generation of NADH. Small portion were utilized for generating acetoin which can avoid excessive acidification of the medium^47^. These suggested that the pyruvate metabolism in *Enterobacter* sp. FY-07 was tended to produce more ATP rather than consume NADH under anaerobic conditions. Deficiency of electron acceptor (oxygen) under anaerobic conditions results in cellular NADH accumulation. To adapt to such circumstances, the microorganism normally regulates certain metabolic pathways to re-oxidise NADH to NAD and supports catabolic mechanisms. Through comparative transcriptome analysis and metabolite quantitation experiment, *Enterobacter* sp. FY-07 was found to upregulate the transcription of genes of the respiratory nitrate reduction pathway and downregulated those of the TCA cycle to eliminate NADH accumulation. In addition, transcription of genes encoding alcohol dehydrogenase and lactate dehydrogenase were not upregulated, which suggested that *Enterobacter* sp. FY-07 cannot re-oxidise NADH to NAD by fermentation to produce ethanol or lactate under anaerobic conditions.

A few *Enterobacter* species have been reported to produce BC, however, it is still little known about their BC biosynthetic machinery^11^,^12^,^16^. Our results demonstrated the BC biosynthetic mechanism, glucose metabolism and energy metabolism of *Enterobacter* sp. FY-07 under aerobic and anaerobic conditions. The present study contributes to the understanding of energy supply mechanism for BC production in facultative anaerobic bacteria under anaerobic conditions.

## Methods

### Strains, plasmids, media and culture conditions

The strains and plasmids used in the study are listed in Table 1. *E. coli* were grown on LB medium (per litre, 10 g peptone, 5 g yeast extract and 5 g NaCl) at 37 °C. *Enterobacter* sp. FY-07 is a highly effective bacterial cellulose (BC) producer that was isolated from a Jilin Oilfield^12^. The resulting culture of FY-07 grown on slant medium (per litre, yeast extract 5 g, peptone 10 g, NaCl 10 g, glucose 10 g and agar 15 g) was washed, re-suspended in sterile water (≈10^7^ cells/ml). The bacterium suspension was inoculated into a 250 ml Erlenmeyer flask containing 100 ml fermentation medium (per litre, NH_4_NO_3_ 2 g, KH_2_PO_4_ 1 g, K_2_HPO_4_-3 H_2_O 0.5 g, MgSO_4_-7 H_2_O 0.25 g, MnCl_2_ 0.2 g, glucose 25 g, pH 7.2) and grown at 30 °C under aerobic conditions. Anaerobic culture was performed as described previously^12^. Briefly, the medium was boiled to remove dissolved oxygen before sterilisation. After sterilisation, filter-sterilised resazurin (1 mg/l) and cysteine hydrochloride (0.5 g/l) were added to the medium. The resulting medium was charged with nitrogen gas until the medium became colourless, after which it was inoculated with the bacterial suspension within an anaerobic chamber. When used for determination of BC production and glucose concentration, the medium inoculated with the bacterial suspension was grown statically at 30 °C. For RNA isolation, the inoculated medium was grown with shaking at 30 °C. Cellulase (1 g/l) was added to the medium for the gene knockout experiment and in the determination of cell growth. A temperature-sensitive (Ts) plasmid named pTSK1, which was constructed from pKD46 (the replication region and *bla* gene) and pEX18Tc (*sac*B, multiple cloning site, Tc and *ori*T) was used for the gene knockout experiment. When needed, the culture was grown at 30 °C for Ts plasmid retention and at 42 °C for Ts plasmid curing. The pBBR1MCS2 plasmid was used for gene complementation. When needed, ampicillin (Ap, 100 μg/ml), tetracycline (Tc, 15 μg/ml), carbenicillin (Car, 50 μg/ml) or kanamycin (Km, 34 μg/ml) were added to the medium.

### DNA extraction and genomic sequencing

Genomic DNA was extracted using a bacterial genomic DNA miniprep kit (Axygen Scientific, Union City, CA, USA), according to the manufacturer’s instructions. Genome sequencing was performed by the Shanghai Majorbio Bio-pharm Biotechnology Company (China). The genome sequencing procedure comprised DNA library preparation, emulsion-based clonal amplification (emPCR), sequencing by the Genome Sequencer FLX system and data synthesis. Firstly, genomic DNA was purified and fragmented to produce small pieces DNA of 400–800 bp. Single-stranded DNA (ssDNA) was recovered after end repair, modification of specific joint connection and denaturation treatment with NaOH. The ssDNA library was fixed on specially designed DNA capture beads to make the most of the beads carrying a unique single-stranded DNA. Secondly, the library bound on the beads was emulsified with amplification reagents to form water-in-oil mixtures. Each bead was amplified independently in their own microreactor. Subsequently, bead-immobilised clonally amplified DNA fragments were recovered and purified for Roche 454 and Solexa sequencing experiments^48^. Gap closing was performed using PCR and a 3730 sequencer. The genome was constructed from 1.2 G 454 reads, 0.8 G Solexa reads and gap-closing reads. The complete genome sequence was analysed by gene prediction and annotation, tRNA/rRNA prediction, COG/KEGG/GO analysis, comparative genome analysis and evolutionary genome analysis. Other methods for DNA manipulation, such as PCR, restriction enzyme reaction and ligation, were performed according to molecular cloning manual^49^ and the manufacturer’s instructions.

### Gene knockout and complementation

To construct the Ts plasmid, the replication unit of pKD46 (≈3000 bp), containing the *rep*A and *bla* genes, was amplified with primerstar pol using the primers p46-1Fw and p46-1Rv (supplementary Table S1). The backbone of pEX18Tc, containing *sac*B, *tet*, *ori*T and multiple cloning site (≈5000 bp), was amplified with primerstar pol using p18-1Fw and p18-1Rv (supplementary Table S1). The resulting fragments were subsequently digested with *Nco*I and *Xho*I and ligated, resulting in pTSK1 (supplementary Fig. S4a).

Gene knockout was performed by homologous recombination. To construct the gene knockout vector, the 1.5 kb upstream and downstream flanking sequences of the candidate gene were PCR amplified from the chromosome of *Enterobacter* sp. FY-07. The DNA fragments were connected by overlap PCR^50^. The resulting fragment was treated with restriction enzymes and then ligated into pTSK1 treated with the same enzymes to generate the gene knockout vector. The gene knockout vector was introduced into *Enterobacter* sp. FY-07 (Car^r^) through conjugation transfer^51^. Car^r^ and Tc^R^ transformants of *Enterobacter* sp. FY-07 were distinguished by PCR using p18-2Fw and p18-2Rv primers (supplementary TableS1). The correct transformant was incubated in LB medium at 37 °C overnight for single-crossover and plasmid curing. The cultures were spread onto LB agar with Tc using the gradient dilution method and incubated overnight at 42 °C. The correct single-crossover colony was identified by PCR and then incubated in LB medium overnight before being spread onto LB plates containing 5–10% sucrose. Two PCR reactions were performed to verify whether the colonies grown on LB with 5–10% sucrose plates were correct or not. The design principle of primers using for verifying the gene knockout mutants is shown in supplementary Fig. S4b. The successful gene knockout strain was complemented by introducing the vector with the amplified corresponding gene ligated into pBBR1MCS2. Cultures of *Enterobacter* sp. FY-07, the gene knockout mutant and its complement were spotted on the LB agar containing 20 mg/L Congo red, which has a strong affinity to cellulose. The degree of staining with this dye was used to estimate cellulose production ability.

### Functional reconstitution of *bcs*III operon in *E. coli* DH5a

The *bcs*III operon and the gene upstream of *bcs*III operon encoding hypothetical protein (AKI40_0895-AKI40_0891) were amplified using *Enterobacter* sp. FY-07 genome as the template. The upstream fragment including the original ribosomal binding site (RBS) sequence was amplified and inserted between the *EcoR*I and *Hind*III sites of the pBAD30^52^, yielding the *bcs*III-expressing vector. The expression of *bcs*III operon was induced by 0.2% L-arabinose at a cell density of OD600 0.5–0.7. Cells were further cultured at 30 °C for 4–5 h. The cells and synthesized cellulose were harvested by centrifugation at 5000 g for 10 min. The precipitate cells and cellulose were fixed with 2.5% glutaraldehyde for overnight, washed by PBS buffer, and then dehydrated for scanning electron microscopy observation.

### RNA isolation

*Enterobacter* sp. FY-07 cultures were collected, immediately frozen in liquid nitrogen and powdered to extract total RNA using Purezol reagent combined with RNAprep pure Cell/Bacteria Kit (Tiangen Biotech, Beijing, China), according to the manufacturer’s protocol. The cultures, harvested by centrifugation at the late logarithmic growth phase under aerobic and anaerobic conditions, were used to prepare transcriptome sequencing RNA samples. Residual genomic DNA contamination was removed by on-column DNase digestion (Takara, Dalian, China), according to the manufacturer’s instruction. RNA integrity was assayed by agarose gel electrophoresis. Sample concentration and quality were quantified at OD260 nm, OD280 nm and OD230 nm by a Bio-drop nano-spectrophotometer.

### Transcriptome sequencing and comparative transcriptome analysis

Total RNA (>5 μg/sample) was used to construct the library and for RNA sequencing. The RNA was treated with the Ribo-zero magnetic Kit (G^+^/G^−^ Bacteria) (Epicentre, USA) to remove rRNA. mRNA enriched RNA was isolated using a Truseq^TM^ RNA sample prep Kit (Illumina, USA), according to the user’s manual. Double stranded cDNA was generated using the double-stranded cDNA Synthesis Kit (Invitrogen, Carlsbad, CA) and ligated with Illumina adaptors. dUTP, instead of dTTP was used to synthesise the second strand. Uracil-N-glycosylase (UNG) was added to digest the second strand cDNA. The library was enriched by 15 cycles of PCR, and recovered from 2% agarose gels. Paired-end 100 bp reads were generated by sequencing using the Hiseq2500 sequencing platform. RNA-Seq reads were mapped to the *Enterobacter* sp. FY-07 reference genome [GenBank accession number CP012487], using the Burrows-Wheeler Aligner^53^. Raw reads data were determined on a per-gene basis and normalised against dividing by a size factor for each library. The normalised read counts, which were divided by the length of gene in kb pairs, were compared with the qRT-PCR data.

Pair-wise differential expression analysis between aerobic and anaerobic conditions was performed using the R package DESeq^54^. An unbiased variance estimator and a negative binomial model were used to test for differential expression by DESeq. To control the false discovery rate, the resulting p values were adjusted using the Benjamini and Hochberg procedure^55^. Genes with a fold-change greater than 2 and an adjusted p value < 0.1 were determined as differentially expressed. The differentially expression genes were classified according to functional categories in the prokaryotic orthologous groups (COG) database (http://www.ncbi.nlm.nih.gov/COG). Genes were determined to be significantly differentially expressed with a selection threshold of fold change (log_2_^anaerobic^/log_2_^aerobic^) ≥2 (group1), ≤2 and ≥1 (group 2), ≤−2 (group 3) and ≤−1 and ≥−2 (group4). Genes associated with specific pathways were analysed using the Kyoto Encyclopedia of Genes and Genomes (KEGG) database (http://www.genome.jp/kegg/).

### Quantitative real-time reverse transcription PCR (qRT-PCR)

qRT-PCR was carried out to access the RNA-sequencing results for the genes of interest. cDNA was synthesised using a Quantscript RT Kit (Tiangen, Beijing, China), following the manufacturer’s instructions. A total input of 500 ng of RNA and random hexamers were used in each reaction. The 16 S rRNA gene was used as the endogenous reference gene. The ratio of expression was quantified by 2^−ΔΔCT^ method. The error of the R-values (ΔR) was calculated by the Bio-rad qPCR software.

### Quantitative methods

Cell growth was measured by monitoring the OD600 nm of cultures using an ultraviolet and visible spectrophotometer at 3-h intervals. After centrifugation, the supernatant of the fermentation broth was filtered by passage through a 0.22-μm membrane (Millipore) and applied to determine the concentration of glucose, sulphate, nitrate, nitrite, formic acid, acetic acid and lactic acid. The supernatant of the culture was extracted by ethyl-acetate, filtered by passage through a 0.22-μm membrane and then applied to determine the concentration of ethanol and acetoin. The concentration of glucose was quantified by the 3, 5-dinitrosalicylic acid (DNS) method, as described previously^56^. Ion chromatography was used to measure the concentration of sulphate, nitrate and nitrite^57^. High performance liquid chromatography (HPLC) was used to measure the concentration of formic acid, acetic acid and lactic acid^58^. Gas chromatography (GC) was applied to quantify the concentration of ethanol and acetoin^59^. To purify BC, the formed BC pellicles were washed, boiled in 1% NaOH(w/v) solution for 10 min, immersed in it for 24 h and then washed with distilled water until the PH of the water was neutral^12^. The purified BC was dried to a constant weigh at 80 °C to measure the BC production. In the determination of BC production, all experiments were performed in triplicate cultures and the relative error of the replicates was less than 5%.

## Additional Information

**How to cite this article**: Ji, K. *et al.* Bacterial cellulose synthesis mechanism of facultative anaerobe *Enterobacter* sp. FY-07. *Sci. Rep.* **6**, 21863; doi: 10.1038/srep21863 (2016).

## Supplementary Material

Supplementary Information

## Figures and Tables

**Table 1:** Strains and plasmids used in this study.

strains	description	reference
*E.coli* s17	*rec*A pro *hsd*R RP4-2-Tc::Mu-Km::Tn7	This lab
*E.coli* DH5α	F^−^φ80 *lac*ZΔM15Δ(*lac*ZYA-*arg*F)U169 *end* A1 *rec*A1 *hsd*R17(rk ^−^, mk ^−^) *sup* E44 λ^−^ thi-1 *gyr*A96 *rel*A1 *pho*A	This lab
*Enterobacter* sp. FY-07	wild-type strain	Ting Ma *et al.*^12^
*Enterobacter* sp. FY-07 Δ*bcs*I	Δ*bcs*I deletion derivative of FY-07	This work
*Enterobacter* sp. FY-07 Δ*bcs*II	Δ*bcs*II deletion derivative of FY-07	This work
*Enterobacter* sp. FY-07 Δ*bcs*III	Δ*bcs*III deletion derivative of FY-07	This work
*Enterobacter* sp. FY-07 Δ*bcs*GFE	Δ*bcs*GFE deletion derivative of FY-07	This work
*Enterobacter* sp. FY-07 Δ*bcs*A(I)	Δ*bcs*A(I) deletion derivative of FY-07	This work
*Enterobacter* sp. FY-07 Δ*bcs*A(II)	Δ*bcs*A(II) deletion derivative of FY-07	This work
*Enterobacter* sp. FY-07 Δ*bcs*A(III)	Δ*bcs*A(III) deletion derivative of FY-07	This work
*Enterobacter* sp.FY-07 Δ*bcs*C(II)	Δ*bcs*C(II) deletion derivative of FY-07	This work
*Enterobacter* sp.FY-07 Δ*hyp*	ΔAKI40_0895 deletion derivative of FY-07	This work
*E.coli* DH5α-pBABIII	*E.coli* DH5α containing pBAD-30-AKI40_0895~AKI40_0891	This work
plasmids		
pKD46	Ts; Amp^r^	Kirill A *et al.*^60^
pEX18Tc	sacB; Tc^r^; oriT	Tung T *et al.*^61^
pTSK1	Ts; sacB; Amp^r^; Tc^r^; oriT	This work
pBBR1MCS2	oriT; Km^r^	Michael E *et al.*^62^
pBAD30	Amp^r^; Para	Guzman L.M. *et al.*^49^
