# Supplementary material for: Bacterial cellulose synthesis mechanism of facultative anaerobe Enterobacter sp. FY-07
Source: Sci Rep. 2016 Feb 25;6:21863. doi: 10.1038/srep21863 (PMC4766428; doi:10.1038/srep21863)
Supplement: Supplementary Information [file srep21863-s1.pdf]

Bacterial cellulose synthesis mechanism of facultative anaerobe *Enterobacter* sp. FY-07

Kaihua Ji[1 +], Wei Wang[2, 3 +], Bing Zeng[1], Sibin Chen[1], Qianqian Zhao[4], Yueqing Chen[1], Guoqiang Li[1*] and Ting Ma[1*]

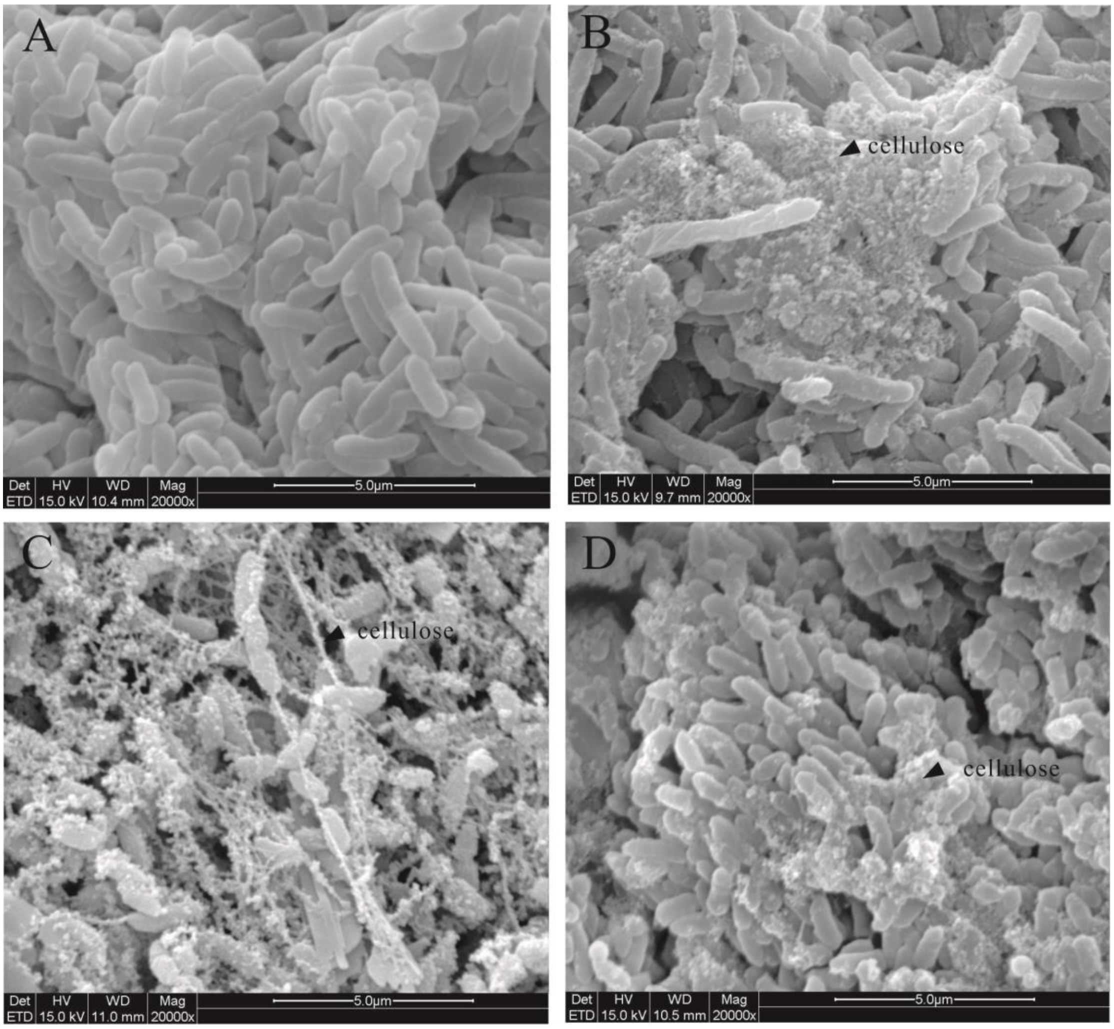

Figure S1: Microscopic observations of *E. coli* transformant cells (A:without inducer; B: with inducer), *Enterobacter* sp. FY-07 (C) and *Enterobacter* sp. FY-07 Δ *hyp* (D).

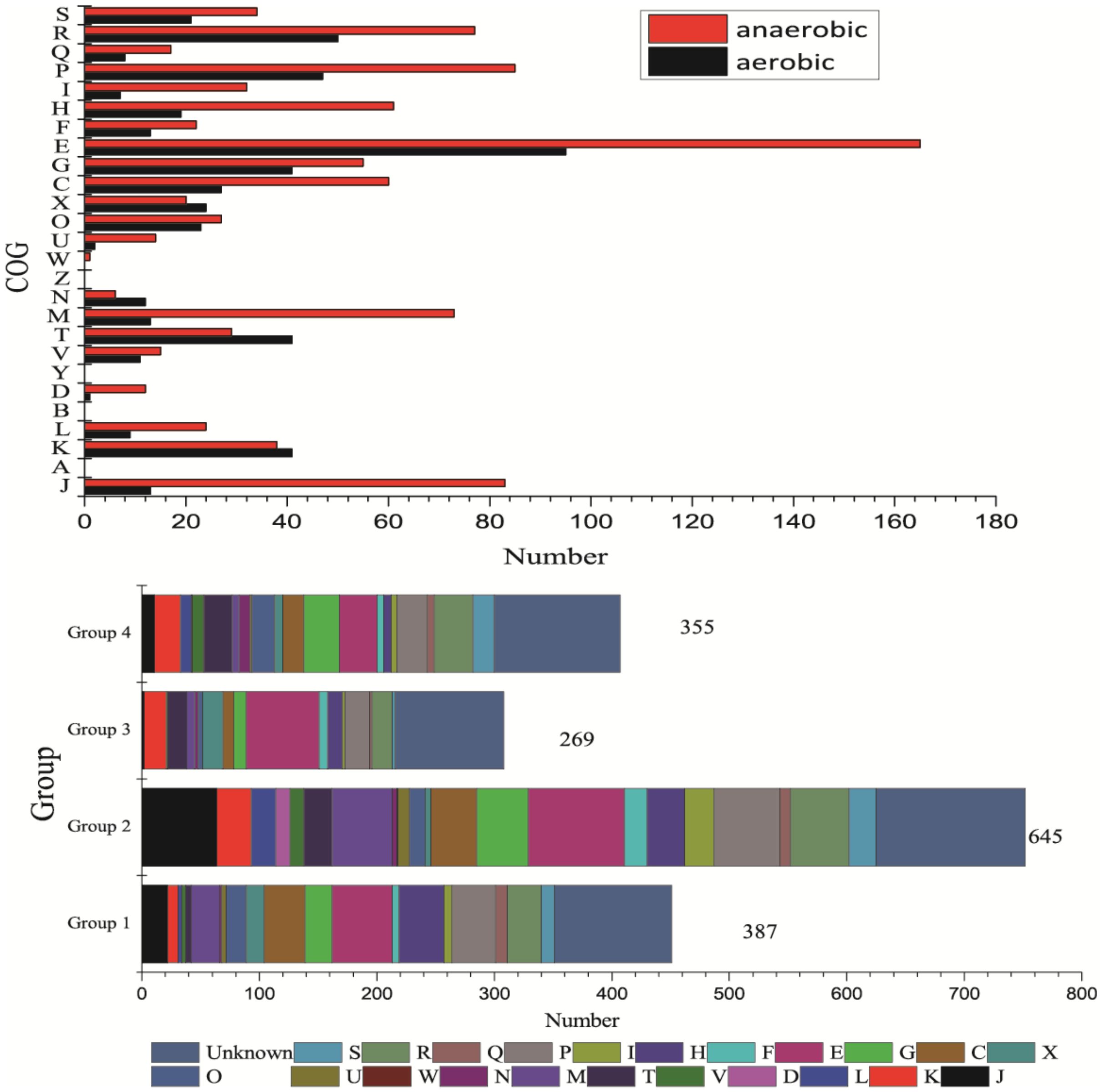

Figure S2: Comparison of transcriptome in identified COG categories of *Enterobacter sp.* FY-07 under aerobic and anaerobic conditions. S function unknown, R general function prediction only, Q secondary metabolites biosynthesis, P inorganic ion transport and metabolism, I lipid transport and metabolism, H coenzyme transport and metabolism, F nucleotide transport and metabolism, E amino acid transport and metabolism, G carbohydrate transport and metabolism, C energy production and conversion; X prophages, transposons, O post-translational modification, protein

turnover, chaperones, U intracellular trafficking and secretion, W extracellular structures, N cell motility, M cell wall/ membrane biogenesis, T signal transduction mechanisms, V defense mechanisms, D cell cycle control, mitosis, and meiosis, L replication, recombination, and repair, K transcription, J translation.

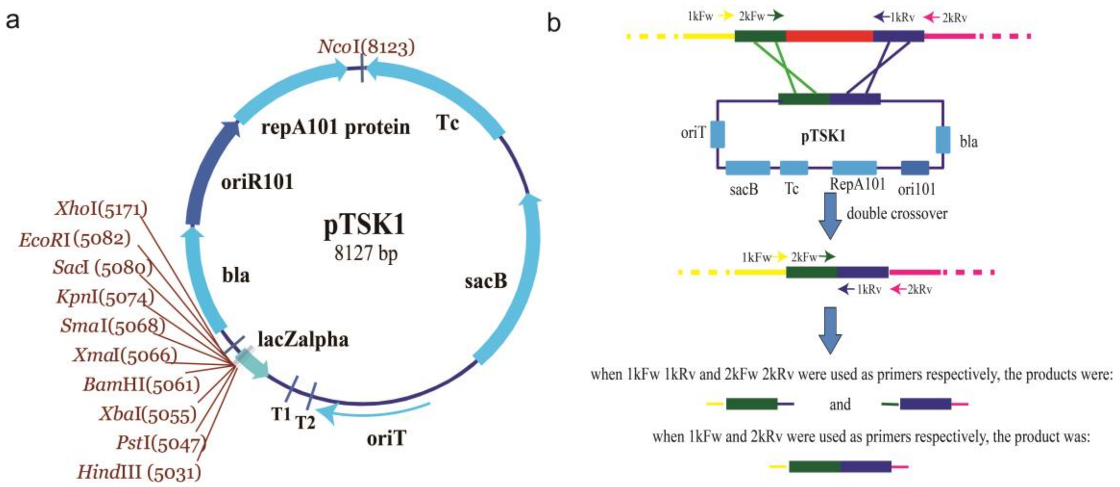

Figure S3: a: plasmid map of temperate-sensitive vector pTSK1 used for gene knockout experiment. b: design strategies of primers used for identification of gene knockout mutants.

Table S1: primers used for constructing of pTSK1 and constructing and identifying of *Enterobacter* sp. FY-07 gene knockout mutants

primer name	sequence(5'-3')	purpose
p46-1Fw	CAGACGAAGAATCCATGGGT	construction of pTSK1
p46-1Rv	GTTCTCGAGATAAAGCGATGCAGGTGGC	
p18-1Fw	TATCCATGGCGGCTTCCATTCAGGTCG	
p18-1Rv	TATCTCGAGGGCACCCCAGGCTTTACA	
bcsI-1Fw	GATCAAGCTTTGTCGGTCGTTGAGCACATTG	construction of gene
bcsI-1Rv	CATTCACCCAGGCAGGCGTCCTTTCGGCGAAC<br>AAACACAAA	knockout vector<br>pTSK-Δ*bcs*I
bcsI-2Fw	TTTGTGTTTGTTCGCCGAAAGGACGCCTGCCT<br>GGGTGAATG	
bcsI-2Rv	TACTAAGCTTATTCCGGCGCAGACTGCTC	
bcsI-1kFw	CGGCCTGATGCTCGACCC	verification of
bcsI-1kRv	CGCCAGCCACACCAGCAT	Enterobacter sp. FY-07
bcsI-2kFw	GCCGGTGATGCAAATGCC	Δ*bcs*I
bcsI-2kRv	CGGTCCGGCCTTCTCGTT	
bcsA(I)-1Fw	TGTAGAATTCAGATCATGGCGGTGGTGGG	construction of gene
bcsA(I)-1Rv	GGTTTCGGCGGGTGAATCCTGGCTGTCCATCG<br>GCGTAA	knockout vector<br>pTSK-Δ*bcs*A(I)
bcsA(I)-2Fw	TTACGCCGATGGACAGCCAGGATTCACCCGCC<br>GAAACC	
bcsA(I)-2Rv	TGTAAAGCTTCAGCGCGCCCTGCTTGTTA	
bcsA(I)-k1Fw	AGCGGCGCTACAATTTCAAC	verification of
bcsA(I)-k1Rv	GCGCGATGTCCAGGTCAT	Enterobacter sp. FY-07
bcsA(I)-k2Fw	GGCACCCCCATGAAAAAGTT	Δ*bcs*A(I)
bcsA(I)-k2Rv	CAATGCGGCTTTTGATGTTGTTA	
bcsII-1Fw	AGCGGAATTCAATATGGTTCAGTGCGGTGTCG	construction of gene
bcsII-1Rv	CGATTAACCACCAGGAATTGTCTTTTAGTTTAA<br>CCGATCCCTATGAATAAAAAGC	knockout vector<br>pTSK-Δ*bcs*II
bcsII-2Fw	GCTTTTTATTCATAGGGATCGGTTAAACTAAAA<br>GACAATTCCTGGTGGTTAATCG	
bcsII-2Rv	GCCCAAGCTTAAATCGCAAATTTTCCCTGCTCA	
bcsII-1kFw	GCGGTGCCATCGTGTTCT	verification of
bcsII-1kRv	GGTACGATAAGCGCAGAGGAAA	Enterobacter sp. FY-07
bcsII-2kFw	AATATGGGGTCCACGATGTCC	Δ*bcs*II
bcsII-2kRv	AGGACTCTTCCAGCACCCAGT	
bcsA(II)-1Fw	GCGCAAGCTTTTTTCCTGATTTGGCATGGTGAA<br>C	construction of gene<br>knockout vector
bcsA(II)-1Rv	GAACAGGCTTTCCAGCGGTTTATGATCAGCAA<br>CCAGGCAGACAGG	pTSK-Δ*bcs*A(II)

bcsA(II)-2Fw	CCTGTCTGCCTGGTTGCTGATCATAAACCGCTGGAAAGCCTGTTC	
bcsA(II)-2Rv	GCCGAAGCTTGGTTGATTGCACCTGCTCTTCATA	
bcsA(II)-k1Fw	ACCAGGCAACGTCATAAACATGT	verification of
bcsA(II)-k1Rv	GCTGGCCCTTGCGTGAC	Enterobacter sp. FY-07
bcsA(II)-k2Fw	GCGTGCGGGCAGTGAG	Δ*bcs*A(II)
bcsA(II)-k2Rv	CAGATCCGGCATGGCAATAAAGT	
bcsIII-1Fw	AGCTGAATTCTCATCGCCGCCAGGTTATCT	construction of gene
bcsIII-1Rv	GCCGGGGTACCTTTATTATTTTCTTTTCGCAAGGGCATTTCCAC	knockout vector pTSK-Δ*bcs*III
bcsIII-2Fw	GTGGAAATGCCCTTGCGAAAAGAAAATAATAAAGGTACCCCGGC	
bcsIII-2Rv	GCTGAAGCTTGAAAGTTTAGTGACACGCTCCTGG	
bcsIII-1kFw	TGTCTGACGGAGCCTCATC	verification of
bcsIII-1kRv	GACTAATTTGATGGCCAGCAGA	Enterobacter sp. FY-07
bcsIII-2kFw	TCATGGCTAAAACCGGTGAGC	Δ*bcs*III
bcsIII-2kRv	GATGGCCACCAGCACGTTATC	
bcsIII-CFw	TAAGGAATTCTACCGTTCAGCCATGTGGG	complementation of
bcsIII-CRv	GCAGTCTAGATTATTCCCCAATGGCGTAACG	Enterobacter sp. FY-07 Δ*bcs*III
bcsA(III)-1Fw	GGACAAGCTTAAAGTTTAGTGACACGCTCCTGGTC	construction of gene knockout vector
bcsA(III)-1Rv	CTGAGGCCCAGTTCGATTTGTAATAAAGGTACCCCGGCATAACAA	pTSK-Δ*bcs*A(III)
bcsA(III)-2Fw	TTGTTATGCCGGGGTACCTTTATTACAAATCGAACTGGGCCTCAG	
bcsA(III)-2Rv	GGCTGAATTCCGGAACGGCAAACGATTTT	
bcsA(III)-k1Fw	GAGCGCATCGTTAACGTCTTT	verification of Enterobacter sp. FY-07
bcsA(III)-k1Rv	GCGCGGGAGAGTACGAC	Δ*bcs*A(III)
bcsA(III)-k2Fw	GTAACCGGAAACATTGTTATGCC	
bcsA(III)-k2Rv	CGGCGCCAGTAGACCTTCT	
bcsA(III)-CFw	TAAGGAATTCTACCGTTCAGCCATGTGGG	complementation of
bcsA(III)-CRv	GCCGTCTAGATTAAAGCGCATTGTTAACCTCTTTC	Enterobacter sp. FY-07 Δ*bcs*A(III)
GFE-1Fw	TATCAAGCTTGGCGATTCCGGTCTCGTTT	construction of gene
GFE-1Rv	GGGCCACGATCTGCATAAAATT GCGAACGCGGTGGTTATTC	knockout vector pTSK-Δ*bcs*GFE
GFE-2Fw	GAATAACCACCGCGTTCGC AATTTTATGCAGATCGTGGCCC	
GFE-2Rv	AGCTAAGCTTGTCGAAAGGGCGTGGTTTG	

GFE-k1Fw	GCTGGTGTTCGGCGTTTTG	verification of
GFE-k1Rv	GGCGGCGTCTGGTGGATAA	Enterobacter sp. FY-07
GFE-k2Fw	GGCGAGGGGAAGTCTGCAG	Δ*bcs*GFE
GFE-k2Rv	TATGCGCCAGCTCTTTTTTGTG	

Table S2: Overview of transcriptome sequencing results and QPCR results of differentially expressed genes

Genebank number	Product	FPKM aerobic	FPKM anaerobic	T-log2 (fold-change) anaerobic/aerobic	Q-log2 (fold-change) anaerobic/aerobic
AKI40_1504	Glucokinase	200.836	194.652	-0.0451201	-0.51
AKI40_4682	Glucose-6-phosphate isomerase	431.923	889.616	1.04241	0.98
AKI40_4866	6-phosphofructokinase isozyme I	970.056	1050.45	0.114871	0
AKI40_2744	6-phosphofructokinase II	124.113	164.152	0.403378	0.47
AKI40_0828	Fructose-bisphosphate aldolase class II	3424.04	6032.63	0.817087	3.04
AKI40_1706	Fructose-bisphosphate aldolase class 1	277.359	394.125	0.506899	0.6
AKI40_4862	Triosephosphate isomerase	1261.65	2804.8	1.15258	1.49
AKI40_3256	Glyceraldehyde-3-phosphate dehydrogenase	638.932	140.947	-2.18051	-1.2
AKI40_0827	Phosphoglycerate kinase	2177.94	3457.79	0.66689	2.09
AKI40_2201	Phosphoglycerate mutase 1 family	1460.27	430.717	-1.76142	0.9
AKI40_0137	Phosphoglycerate mutase	199.618	994.072	2.31611	1.97
AKI40_4275	Probable phosphoglycerate mutase gpmB	383.729	233.797	-0.71483	0.65
AKI40_4050	Enolase	2972.58	8704.85	1.55011	3.08
AKI40_2849	Pyruvate kinase I	909.613	1585.6	0.801704	1.25
AKI40_3571	Pyruvate kinase II	288.492	299.764	0.0552963	-1.16
AKI40_4472	Fructose-1,6-bisphosphatase class 1	771.316	932.455	0.273713	1.82
AKI40_1324	Archaeal fructose-1,6-bisphosphatase and related enzymes of inositol monophosphatase family	208.354	1056.79	2.34259	2.06
AKI40_4858	Fructose-1,6-bisphosphatase class II	93.8551	207.062	1.14156	2.99
AKI40_2388	Formate acetyltransferase	1278.86	4079.42	1.67351	2.52
AKI40_4164	Dihydrolipoyl dehydrogenase	524.198	789.516	0.590856	0.87

AKI40_4165	Dihydrolipoyllysine-residue acetyltransferase component of pyruvate dehydrogenase c	318.706	909.033	1.51211	4.49
AKI40_4166	Pyruvate dehydrogenase E1 component	482.222	1012.98	1.07083	
AKI40_4167	Pyruvate dehydrogenase complex repressor (GntR-family transcriptional regulator)	1179.58	2366.48	1.00447	3.57
AKI40_1562	Phosphate acetyltransferase (Phosphotransacetylase)	121.343	1064.64	3.1332	2.41
AKI40_1564	Acetate kinase A and propionate kinase 2	314.873	1131.53	1.84543	4.12
AKI40_2176	Citrate synthase	458.263	175.657	-1.38342	-2.29
AKI40_2177	Succinate dehydrogenase hydrophobic membrane anchor protein	61.2079	28.3785	-1.10892	
AKI40_2179	Succinate dehydrogenase flavoprotein subunit	69.0544	25.9928	-1.40962	-4.9
AKI40_2180	Succinate dehydrogenase iron-sulfur protein	151.322	60.3974	-1.32506	
AKI40_2181	Oxoglutarate dehydrogenase, E1 component	124.205	98.6758	-0.331957	-4.9
AKI40_2182	Dihydrolipoyllysine-residue succinyltransferase, E2 component of oxoglutarate dehydrogenase complex	200.024	236.902	0.244116	-2.41
AKI40_2183	Succinyl-CoA ligase subunit beta	302.695	257.912	-0.230989	-1.59
AKI40_2184	Succinyl-CoA ligase subunit alpha	343.033	305.514	-0.167113	-2.56
AKI40_3315	Aconitate hydratase 1	97.2913	65.6526	-0.567459	-4.94
AKI40_4162	Aconitate hydratase 2	359.791	267.305	-0.428672	-3.66
AKI40_2659	Isocitrate dehydrogenase	1018.27	592.589	-0.781019	-3.16
AKI40_3457	Fumarate reductase, flavoprotein subunit	61.4202	26.6241	-1.20598	-4.34
AKI40_4544	Fumarate reductase, flavoprotein subunit'	159.534	281.152	0.817487	
AKI40_4545	Fumarate reductase iron-sulfur subunit	184.04	381.869	1.05306	-2.32

AKI40_4546	Fumarate reductase subunit C	157.783	253.803	0.685766	
AKI40_4547	Fumarate reductase subunit D	249.139	360.491	0.533009	
AKI40_3459	Tartrate/fumarate subfamily Fe-S type hydro-lyase beta subunit	26.5898	137.729	2.37289	-2.59
AKI40_0551	Malate dehydrogenase	424.937	72.4416	-2.55236	-3.83
AKI40_3010	Malate dehydrogenase	80.0799	30.0939	-1.41197	-3.78
AKI40_1562	Phosphate acetyltransferase	121.343	1064.64	3.1332	0.89
AKI40_1564	Acetate kinase A and propionate kinase 2	314.873	1131.53	1.84543	2.99
AKI40_2388	Formate acetyltransferase 1	1278.86	4079.42	1.67351	1.11
AKI40_3156	Formate dehydrogenase gamma subunit	19.0707	862.409	5.49894	
AKI40_3157	Formate dehydrogenase, beta subunit'	9.04282	643.461	6.15294	
AKI40_3158	Formate dehydrogenase alpha subunit	6.13931	648.073	6.72193	7.23
AKI40_3242	Putative formate dehydrogenase	57.9734	83.5321	0.52694	0.98
AKI40_4598	Formate dehydrogenase H	30.0649	94.421	1.65103	
AKI40_4599	Formate dehydrogenase H alph subunit	38.5238	111.252	1.53001	1.35
AKI40_4203	Acetolactate synthase 3 regulatory subunit	266.47	435.775	0.70961	
AKI40_4204	Acetolactate synthase isozyme 3 large subunit	151.707	185.13	0.287251	0.87
AKI40_4818	acetolactate synthase 2 catalytic subunit	234.177	238.956	0.0291437	0.74
AKI40_0023	acetolactate synthase catalytic subunit	45.622	68.8787	0.59433	0.22
AKI40_0024	Acetolactate synthase, isozyme I, small subunit	59.785	92.0479	0.622601	
AKI40_0778	Alpha-acetolactate decarboxylase	11.7533	162.944	3.79324	1.45
AKI40_0779	Acetolactate synthase, catabolic	16.2135	109.176	2.75139	1.26
AKI40_2159	Phosphoglucomutase, alpha-D-glucose phosphate-specific	831.796	957.021	0.202321	

AKI40_3569	Glucose-6-phosphate 1-dehydrogenase	570.713	485.82	-0.232344	0.36
AKI40_2213	6-phosphogluconolactonase	677.087	740.52	0.129199	0.31
AKI40_1765	6-phosphogluconate dehydrogenase, decarboxylating	671.096	1128.29	0.749545	
AKI40_0415	Ribulose-phosphate 3-epimerase	221.896	283.861	0.3553	0.83
AKI40_0833	Ribose-5-phosphate isomerase A	145.176	213.574	0.556938	-2.15
AKI40_0825	Transketolase 1	92.6359	772.757	3.06037	2.77
AKI40_1411	Transketolase 2	137.617	299.799	1.12334	-2.95
AKI40_4259	transaldolase B	844.218	1161.07	0.459773	3.31
AKI40_1412	transaldolase A	212.188	347.861	0.713169	-2.41
AKI40_1025	Nitrate ABC superfamily ATP binding cassette transporter, binding protein	1159.98	12.5279	-6.53281	-3.01
AKI40_3367	Respiratory nitrate reductase gamma subunit	28.8085	3302.72	6.84102	
AKI40_3368	Nitrate reductase molybdenum cofactor assembly chaperone 1	45.7939	4507.69	6.62109	
AKI40_3369	Nitrate reductase 1, beta subunit	44.5786	3714.97	6.38086	
AKI40_3370	Nitrate reductase 1, alpha subunit	193.523	2459.45	3.66776	3.93
AKI40_0419	Probable nitrite transporter	26.0375	889.012	5.09354	
AKI40_0420	Nitrite reductase small subunit	117.292	2605.09	4.47316	
AKI40_0421	Nitrite reductase, large subunit	360.343	2075.99	2.52636	7.38
AKI40_4912	Glutamine synthetase, type I	5481.98	7589.12	0.469235	0.47
AKI40_2718	Glutamate dehydrogenase	8.35574	216.797	4.69744	0.34
AKI40_0561	Glutamate synthase, small subunit	215.965	1875.86	3.11868	2.05
AKI40_0563	Glutamate synthase large subunit	200.788	1437.91	2.84023	4.19
AKI40_3245	Alcohol dehydrogenase class III	162.556	96.0072	-0.759722	-2.36

AKI40_3351	Fused acetaldehyde-CoA dehydrogenase iron-dependent alcohol dehydrogenase pyruvate-formate lyase deactivase	1166.75	1495.56	0.358197	-1.02
AKI40_4427	Alcohol dehydrogenase GroES domain protein	125.681	170.345	0.438694	-1.45
AKI40_1686	D-lactate dehydrogenase, membrane binding family	229.141	263.827	0.203353	-1.16
AKI40_1574	NADH-quinone oxidoreductase subunit B	446.818	763.856	0.773613	0.75
AKI40_1576	NADH-quinone oxidoreductase subunit E	407.424	1086.11	1.41457	1.11
AKI40_1578	NADH-quinone oxidoreductase subunit G	139.616	800.381	2.51922	0.18
AKI40_1580	NADH-quinone oxidoreductase subunit I	122.935	412.423	1.74623	1.51
AKI40_1582	NADH-quinone oxidoreductase subunit K	99.1131	508.929	2.36032	1.68
AKI40_1584	Proton-translocating NADH-quinone oxidoreductase, chain M	197.545	593.989	1.58825	0.25
AKI40_2908	NADP transhydrogenase alpha subunit	208.479	590.045	1.50093	
AKI40_2909	NAD/NADP transhydrogenase beta subunit	241.184	860.88	1.83568	2.76
AKI40_0155	Predicted hydrogenase, 4Fe-4S ferredoxin-type component	59.2272	133.83	1.17607	1.51
AKI40_0754	hydrogenase 2 small subunit	4.26097	57.0216	3.74226	3.53
AKI40_0755	Hydrogenase 2 4Fe-4S ferredoxin-type component	2.60715	41.0672	3.97744	
AKI40_0756	Ni/Fe-hydrogenase 2 b-type cytochrome subunit	11.289	52.1486	2.20771	
AKI40_0757	Hydrogenase 2 large subunit	8.37577	125.41	3.90429	
AKI40_0758	Maturation element for hydrogenase 2	6.14083	147.646	4.58757	

AKI40_0759	Hydrogenase 2-specific chaperone	4.59081	118.045	4.68445
AKI40_0760	Hydrogenase nickel incorporation protein HybF	16.5955	155.503	3.22808
AKI40_0761	Hydrogenase maturation factor	22.1362	183.359	3.05019
AKI40_1736	GDP-D-mannose dehydratase, NAD binding	484.648	2782.16	2.5212
AKI40_1739	Glycosyl transferase, group 1	92.7938	1519.53	4.03345
AKI40_1745	Colanic acid biosynthesis glycosyl transferase WcaL	95.2427	892.364	3.22795
AKI40_0889	UTP-glucose-1-phosphate uridylyltransferase, GalU protein	1796.25	388.598	-2.20864
AKI40_0890	Diguanylate cyclase/phosphodiesterase with PAS/PAC sensor	1048.97	330.394	-1.66672
AKI40_0894	glycosyl transferase, group 2 family protein	1188.75	335.907	-1.82331
AKI40_0893	Cyclic di-GMP-binding protein, Cellulose synthase operon protein B	866.701	453.901	-0.933155
AKI40_0892	Cellulose synthase operon C domain protein	416.052	532.715	0.356601
AKI40_0891	Hypothetical protein	1511.2	497.382	-1.60327
AKI40_4699	Isocitrate lyase, putative	12.399	13.3457	0.106143
AKI40_4700	Malate synthase A	8.66897	7.4973	-0.209488
AKI40_1747	UTP-glucose-1-phosphate uridylyltransferase subunit GalU	536.68	1793.13	1.74035
AKI40_3354	UTP-glucose-1-phosphate uridylyltransferase subunit GalU	657.817	953.92	0.536182
AKI40_0196	Hypothetical protein	59.5842	135.518	1.18548
AKI40_0197	Cellulose synthase operon protein YhjQ -like protein	45.0796	79.1283	0.811719
AKI40_0198	Cellulose synthase	14.8801	62.6109	2.07303

AKI40_0199	Cellulose synthase, subunit B	11.0953	78.8136	2.82849
AKI40_0200	Cellulose synthase operon C domain protein	9.45997	54.9519	2.53826
AKI40_0201	Cellulose synthase operon protein D	33.1256	74.1816	1.16311
AKI40_0202	Glycosyl hydrolase, family 8	85.8899	131.762	0.617371
AKI40_0206	Predicted protein	182.711	118.749	-0.621648
AKI40_0207	Cellulose synthase operon protein YhjQ	155.105	128.421	-0.272372
AKI40_0208	Cellulose synthase catalytic subunit	69.9293	98.0741	0.487974
AKI40_0209	Cellulose synthase, subunit B	74.2767	181.718	1.29072
AKI40_0210	Endo-1,4-beta-glucanase	53.6828	178.102	1.73017
AKI40_0211	Cellulose synthase operon C domain protein	50.7703	190.288	1.90613
